# A systematic comparison of patient information leaflets from local and multinational pharmaceutical companies: Assessing content quality and completeness

**DOI:** 10.1371/journal.pone.0335149

**Published:** 2025-11-07

**Authors:** Abdulaziz Ibrahim Alzarea, Azfar Athar Ishaqui, Muhammad Zeeshan, Abdullah Salah Alanazi, Aseel Awad Alsaidan, Tauqeer Hussain Mallhi, Muhammad Salman, Yusra Habib Khan, Javeria Farooq, Hassan H. Alhassan, Sami I. Alzarea, Omar Awad Alsaidan

**Affiliations:** 1 Department of Clinical Pharmacy, College of Pharmacy, Jouf University, Sakaka, Saudi Arabia; 2 Department of Clinical Pharmacy, College of Pharmacy, King Khalid University, Abha, Saudi Arabia; 3 Department of Pharmacy, Iqra University, Karachi, Pakistan; 4 Department of Family and Community medicine, College of medicine, Jouf University, Sakaka, Saudi Arabia; 5 School of Pharmacy, Faculty of Health and Medical sciences, Taylors University, Selangor, Malaysia; 6 Medicines R Us Chemist, Gregory Hills, New South Wales, Australia; 7 Institute of Pharmacy, Faculty of Pharmaceutical and Allied Health Sciences, Lahore College for Women University, Lahore, Pakistan; 8 Faculty of Pharmacy, University of Karachi, Karachi, Pakistan; 9 Department of Clinical Laboratory Sciences, College of Applied Medical Sciences, Jouf University, Sakaka, Saudi Arabia; 10 Department of Pharmacology, College of Pharmacy, Jouf University, Sakaka, Saudi Arabia; 11 Department of Pharmaceutics, College of Pharmacy, Jouf University, Sakaka, Saudi Arabia; Universitas Airlangga, INDONESIA

## Abstract

**Background:**

It is necessary to provide essential information to patients regarding medication to ensure safe and effective use. Patient Information Leaflets (PILs) are vital to fulfil this purpose. However, disparities in quality between multinational and local pharmaceutical companies PILs have been observed. The study aimed to assess and compare the completeness and quality of PILs available in community pharmacies from multinational and local pharmaceutical companies.

**Method:**

A cross-sectional comparative study design was used for evaluating 695 PILs (312 multinational, 383 local) across various therapeutic classes (antibiotics, anti-hypertensives, anti-diabetics, NSAIDs, and antidepressants). PILs were assessed against 31 criteria encompassing general information, dosage, indications, administration, interactions, safety information, pharmacokinetics, toxicology, storage, adverse effects, and pregnancy/breastfeeding details. Descriptive statistics, t-tests, chi-square tests, and odds ratios were included in statistical analysis.

**Result:**

Several criteria results showed excelling of Multinational PILs over local PILs. Significant differences favouring multinational PILs were observed in providing information on inactive ingredients (p = 0.024), adult-specific dosing (p = 0.0001), renal and hepatic dose adjustments (p = 0.018, p = 0.007 respectively), dosing in haemodialysis patients (p = 0.036), drug-drug interactions (p = 0.029), black box warnings (p = 0.027), clinical effects of toxicity (p = 0.045), and reporting adverse drug reactions (p = 0.04).

**Conclusion:**

Multinational and local PILs, both performed well in providing basic information on active ingredients, brand names, contraindications, pregnancy, and lactation details. However, disparities among various aspects including inactive ingredient, specific dosing, dose adjusments and drug interactions between PILS of multinational and local pharmaceutical companies in Pakistan highlights the necessity for regulatory measures and industry initiatives to standardize PIL content. For ensuring patient safety and enhancing the quality of medication use it is essential to improve the comprehensiveness and clarity of PILs.

## Introduction

The patient information leaflets (PILs) are educational documents intended to aid patients’ regarding the proper use of their medications (e.g. how to use the drugs, dosage, potential side effects, contraindications and other essential information), thus, improving patients’ awareness and ensuring the efficacy and safety of the used medicines. The PILs also helps in enforcing the recommended treatments among patients [1]. The provision of written information to patients has its roots in the early 20^th^ century, and the first medication leaflets were prepared, including basic information about the drugs. However, PILs did not assume a significant amount of standard form and popularity until the 1960s and 1970s due to consumer rights movements and legislation development. In 1977, the Food and Drug Administration (FDA) of the United States asserted that prescription drugs should contain information that is easily understandable for patients, a decisive move towards creating PILs. The European Union also incorporated similar conditions during the 1990s and solidified the practice with even more regulations. Although the PILs are supposed to contain all the relevant information, failure to include specific information in PILs happens due to lack of regulatory standards for preparing PILs. Such variation results in significant gaps within the information delivered to patients and safe ways of using the medications [2]. The contradiction in the provided information can be misleading for patients, creating non-adherence and adverse health consequences [3]. A retrospective study conducted in Sri Lankan hospitals revealed that most PILs were of poor quality and did not meet standard references, leading to medication errors [4].

PILs for different brands of a medicine should be comparable in terms of the content, clarity, comprehensibility and completeness. Although, the multinational corporations (MNCs) create very elaborate PILs, this is not case with the local manufacturers in low-middle-income countries. For instance, in a survey conducted on PILs of NSAIDs in Palestine, the details provided in the imported PILs was better compared to the local PILs.[5] Similarly, Eshtayeh et al. [6] reported that the local PILs of oral antidiabetic agents available in Palestine were insufficient and less elaborate than those of the MNCs. Moreover, studies conducted by Qatmosh et al and Sawalha et al on anti-hypertensive and anti-infective drug PILs observed that MNCs PILs superior over local PILs in quantity and quality of information [7,8].

Concerns have been raised about the adequacy of PILs in Pakistan in delivering essential drug-related information to patients. Previous studies have identified significant deficiencies in the completeness and accuracy of PILs revealing critical gaps in content [9,10]. While these studies have highlighted the general shortcomings of PILs in Pakistan, there remains a gap in systematically comparing the quality and completeness of PILs from multinational versus local pharmaceutical companies. The current study aims to address this gap by conducting a comprehensive evaluation of PILs from both sectors, providing insights into potential disparities and areas for regulatory improvement.

## Methodology

### Study design

A cross-sectional study was conducted to assess the content and quality of PILs between multinational and local pharmaceutical products available in the Pakistani market.

### Sample selection

This study evaluated PILs from both multinational and locally manufactured pharmaceutical products in Pakistan, focusing on major therapeutic categories: antibiotics, anti-hypertensive agents, non-steroidal anti-inflammatory drugs (NSAIDs), anti-diabetic agents, anti-psychotics, anti-epileptic and anti-depressants. The selection criteria targeted widely prescribed medications representative of the Pakistani pharmaceutical market, ensuring relevance to clinical practice. To make a proper comparison, only PILs of tablet dosage forms were included in the analysis. Similarly, in the case of any generic drug under investigation; both local and multinational pharmaceutical brands were included. PILs were collected from community pharmacies in Pakistan over a 6-month period from July, 2024 until December, 2024. It was considered necessary to collect at least three leaflets of both multinational and local brands to get a broader comparison of the quality and the amount of information that can be received from the PILs of both local and multinational products.

### Evaluation criteria

The quantitative and qualitative comparison of each PIL was made by giving 31 points of information in 11 categories: (a) General information, (b) Dosage information, (c) Indications, (d) Administration directions, (e) drug Interactions, (f) Pregnancy/breastfeeding information (g) Medication safety information (h) Mechanism/ pharmacokinetics (i) Toxicology (j) Storage and (k) Adverse effects.

### Scoring system

The PILs were assessed against 31 criteria, the component topics of drug information that the PILs should contain. For each criterion, the response was coded as “1” if the information was reported and as “0” if it was not; the total score, therefore, attainable for each PIL, was 31. The total scores were then classified into four categories. A score of 28–31 points was considered as comprehensive or outstanding, 24–27 points as good or satisfactory, 18–23 points as basic or limited, and 0–17 points as inadequate or insufficient.

### Data collection

The study employed a rigorous two-stage review process where two independent researchers evaluated PILs using a 31-item checklist. Initial disagreements were resolved through discussion, with unresolved cases adjudicated by a third senior investigator. This three-tier approach enhanced reliability and minimized bias in the PIL quality assessment.

### Statistical analysis

The data collected were entered and analyzed using the Statistical Package for Social Sciences (SPSS) version 16. In summarizing and comparing the collected data, categorical data were presented as frequency and percentage, whereas continuous data were presented as mean, standard deviation, and median. The t-test was used to compare the mean scores, while the chi-square was used to compare categorical data. To quantify the relationship between the presence of the particular criteria in local and multinational PILs, odds ratios (OR) with 95% confidence intervals (CI) were determined. A p-value of < 0.05 was regarded as statistically significant.

## Results

The study analyzed a total of 695 patient information leaflets (PILs), comprising 312 from multinational pharmaceutical companies and 383 from local manufacturers. These PILs represented seven major therapeutic categories: antibiotics (n = 226), anti-hypertensive agents (n = 120), non-steroidal anti-inflammatory drugs (NSAIDs; n = 74), anti-depressants (n = 77), anti-diabetic medications (n = 69), anti-epileptic drugs (n = 68), and antipsychotics (n = 61). The details of comparisons of the completeness and quality of PILs from these two groups are presented in Table 1.

**Table 1:** Comparison of Medication Information in Multinational vs. Local Patient Information Leaflets.

		Overall	Multi	Local	P-Value	Odds Ratio
Generic						
1	Brand name	695 (100%)	312 (100%)	383 (100%)	–	–
2	Active ingredient	695 (100%)	312 (100%)	383 (100%)	–	–
3	In-active ingredients	359 (51.6%)	176 (56.4%)	183 (47.8%)	0.024	1.41 (1.05- 1.91)
Dosing Information						
4	Adult indication-specific dosing	272 (39.1%)	162 (50.1%)	146 (38.1%)	0.0001	2.38 (1.69- 3.35)
5	Pediatric indication-specific dosing	381 (54.8%)	178 (57.1%)	203 (53%)	0.286	1.18 (0.87- 1.59)
6	Geriatric indication-specific dosing	259 (37.2%)	124 (39.7%)	135 (35.2%)	0.223	1.12 (0.88- 1.65)
7	Maximum dose per day	390 (56.1%)	170 (54.5%)	220 (57.4%)	0.435	0.88 (0.65- 1.19)
8	Dosing in obese patients	67 (9.64%)	29 (9.29%)	38 (9.92%)	0.781	0.93 (0.55- 1.55)
9	Renal dose adjustment mentioned or not	362 (52.1%)	178 (57.1%)	184 (48%)	0.018	1.43 (1.06- 1.94)
10	Hepatic dose adjustment or not	315 (45.3%)	159 (50.9%)	156 (40.7%)	0.007	1.51 (1.1- 2.04)
11	Information regarding use in hemodialysis or not	157 (22.6%)	82 (26.3%)	75 (19.6%)	0.036	1.46 (1.02- 2.09)
Indications						
12	FDA-approved indications	309 (44.5%)	144 (46.2%)	165 (43.1%)	0.417	1.13 (0.83- 1.53)
13	Non-FDA indications	146 (21%)	63 (20.2%)	83 (21.7%)	0.634	0.91 (0.63- 1.32)
Administration Instructions						
14	Information regarding if taken with food or not	391 (56.3%)	180 (57.7%)	211 (55.1%)	0.492	1.1 (0.82- 1.50)
15	If the tablet can be crush or not	69 (9.92%)	43 (13.8%)	26 (6.7%)	0.002	2.19 (1.31- 3.65)
Drug Interactions						
16	Drug-drug interactions	597 (85.9%)	278 (89.1%)	319 (83.3%)	0.029	1.64 (1.05- 2.56)
17	Drug-Lab interactions	207 (29.8%)	102 (32.7%)	105 (27.4%)	0.13	1.29 (0.93- 1.78)
18	Drug-food interactions	197 (28.3%)	91 (29.2%)	106 (27.7%)	0.664	1.07 (0.77- 1.49)
Pregnancy/Lactation Information						
19	Pregnancy category mentioned or not	493 (70.9%)	223 (71.5%)	270 (70.5%)	0.778	1.05 (0.754- 1.46)
20	Clearly mentioned that it can be used in lactation or not	500 (71.9%)	224 (71.8%)	276 (72.1%)	0.937	0.98 (0.71- 1.38)
Medication Safety Information						
21	Black box warning mentioned or not	300 (43.2%)	149 (47.8%)	151 (39.4%)	0.027	1.40 (1.04- 1.90)
22	Contraindications mentioned or not	651 (93.7%)	295 (94.6%)	356 (93%)	0.389	1.31 (0.70- 2.46)
23	Precautions mentioned or not	652 (93.8%)	295 (94.6%)	357 (93.2%)	0.466	1.26 (0.67- 2.37)
24	Adverse effects mentioned or not	609(87.6%)	275 (88.1%)	334 (87.2%)	0.709	1.09 (0.69- 1.72)
25	Monitoring parameters mentioned or not	235 (33.8%)	115 (36.9%)	120 (31.3%)	0.126	1.28 (0.93- 1.75)
Mechanism/ Pharmacokinetics						
26	Mechanism of action	461 (66.3%)	204 (65.4%)	257 (67.1%)	0.633	0.93 (0.68- 1.27)
27	Pharmacokinetics- absorption	473 (68.1%)	214 (68.6%)	259 (67.6%)	0.786	1.04 (0.76- 1.44)
Toxicology						
28	Clinical effects	374 (53.8%)	181 (58.1%)	193 (50.4%)	0.045	1.36 (1.01- 1.84)
29	Treatment of toxicity	251 (36.1%)	126 (40.4%)	125 (32.6%)	0.034	1.39 (1.02- 1.91)
Storage						
30	Related to temperature	576 (82.9%)	260 (83.3%)	316 (82.5%)	0.774	1.06 (0.72- 1.58)
ADR Reporting/ Feedback						
31	Contact information to report any ADR to company	289 (41.6%)	143 (45.8%)	146 (38.1)	0.04	1.37 (1.01- 1.86)

Around 56% of MNCs products leaflets provided information on the inactive ingredients as compared to 47.8% in local brands (p = 0.024; odds ratio (OR):1.41 (95% CI: 1.05–1.91). Multinational PILs were also significantly more likely to include adult indication-specific dosing information (40.4% vs. 38.1%, p < 0.001, OR: 2.38, 95% CI: 1.69–3.35). They also provided more details on the renal dose adjustments (57.1% vs. 48%, p = 0.018, OR: 1.43, 95% CI: 1.06–1.94) and hepatic dose adjustments (50.9% vs. 40.7%, p = 0.007, OR: 1.51, 95% CI: 1.10–2.04). Additionally, the information regarding dosing in hemodialysis patients was also more frequently included in multinational PILs (26.3% vs. 19.6%, p = 0.036, OR: 1.46, 95% CI: 1.02–2.09). Multinational PILs were more comprehensive in listing drug-drug interactions (DDIs; 89.1% vs. 83.3%, p = 0.029, OR: 1.64, 95% CI: 1.05–2.56).

More multinational PILs contained data on the black box warnings compared to local manufacturers’ PILs(47.8% vs. 39.4%, p = 0.027, OR: 1.40, 95% CI: 1.04–1.90). Furthermore, toxicological effects were more frequently detailed in the multinational PILs (58.1% vs. 50.4%, p = 0.045, OR: 1.36, 95% CI: 1.01–1.84), and treatment of toxicity was also better addressed (40.4% vs. 32.6%, p = 0.034, OR: 1.39, 95% CI: 1.02–1.91). We did not observe any significant difference between multinational and local PILs regarding the inclusion of pregnancy category information (71.5% vs. 70.5%, p = 0.778, OR: 1.05, 95% CI: 0.754–1.46) and clear indications about use during lactation (71.8% vs. 72.1%, p = 0.937, OR: 0.98, 95% CI: 0.71–1.38). Lastly, the contact information for reporting any adverse drug reactions (ADRs) was more frequently included in multinational PILs (45.8% vs. 38.1%, p = 0.04, OR: 1.374, 95% CI: 1.01–1.86).

Overall, these findings indicate that the multinational pharmaceutical companies generally provide more comprehensive and detailed information in their PILs than local companies, particularly in critical areas such as inactive ingredients, dosing information, drug interactions, medication safety, and ADR reporting. The higher quality of multinational PILs can significantly enhance patient understanding and safety. Despite the overall trend favoring multinational pharmaceutical companies, certain aspects were well-addressed in both multinational and local PILs. Both groups showed high completeness in listing the active ingredients (100% for both) and brand names (100% for both). Information on contraindications was also robustly provided by both multinational (94.6%) and local (93%) companies, with no significant difference (p = 0.389). Furthermore, multinational and local PILs adequately included pregnancy category information and clear indications about use during lactation. These findings indicate that while there are disparities in other areas, multinational and local PILs generally performed well in providing essential drug information.

Table 2 shows the overall scores of PILs from both multinational and local pharmaceutical companies.

**Table 2:** Grading of Patient Information Leaflets Based on Completeness of Information.

Classification	Overall (n = 695)	Local (n = 383)	Multi (n = 312)	P-Value
28-31 points: Comprehensive or Outstanding	6 (0.86%)	1 (0.26%)	5 (1.60%)	0.004
24-27 points: Good or Satisfactory	69 (9.90%)	26 (6.79%)	43 (13.78%)	
18-23 points: Basic or Limited	229 (32.86%)	120 (31.33%)	109 (34.94%)	
0-17 points: Inadequate or Insufficient	391 (56.10%)	236 (61.62%)	155 (49.68%)	
Overall Mean	16.91	16.53	17.38	0.012
Standard Deviation	4.52	4.61	4.36	
Median	17	16	17	

**Fig 1:**
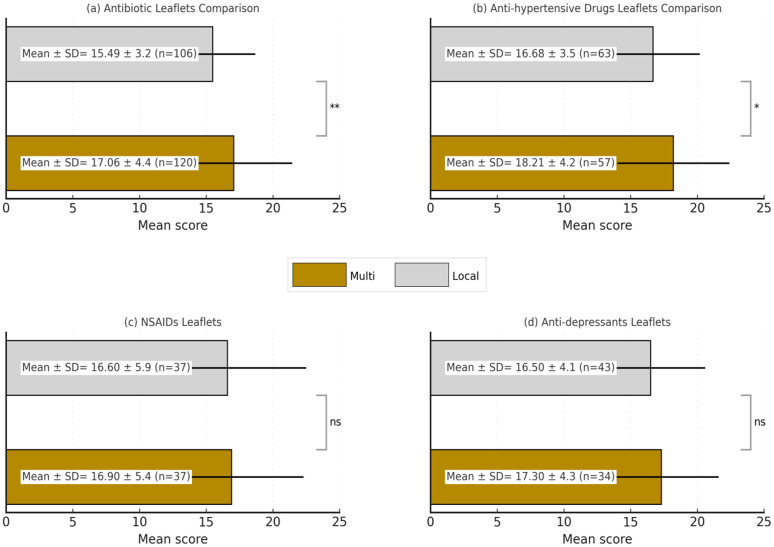
Graphical Comparison of Completeness Scores for Patient Information Leaflets of Different Drug Classes a) Comparison of mean completeness scores for antibiotic patient information leaflets (b) Comparison of mean completeness scores for anti-hypertensive drug patient information leaflets (c) Comparison of mean completeness scores for NSAID patient information leaflets (d) Comparison of mean completeness scores for anti-depressant patient information leaflets. Where: ns = non-significant, * = p-value < 0.05, **= p-value < 0.001.

The classification shows that only 0.86% of all PILs achieved a “Comprehensive or Outstanding” score, with multinational companies accounting for a higher proportion of this category (1.60% vs. 0.26%, p = 0.004). Additionally, 13.78% of multinational PILs were rated as “Good or Satisfactory,” compared to 6.79% of local PILs. A significant portion of PILs fell into the “Inadequate or Insufficient” category, with local companies more frequently represented in this group (61.62% vs. 49.68%, p = 0.004). The mean score for multinational PILs was also significantly higher than for local ones (17.38 vs. 16.53, p = 0.012), indicating a general trend of better quality in multinational PILs. Fig 1 compares the mean scores of PILs for antibiotics, anti-hypertensives, NSAIDs, and antidepressants between multinational (Multi) and local (Local) pharmaceutical companies. The mean score for local antibiotic leaflets was 15.49 ± 3.2 while multinational antibiotic leaflets score significantly higher at 17.06 ± 4.4 (p < 0.01). For anti-hypertensives, local leaflets had a mean score of 16.68 ± 3.5) compared to 18.21 ± 4.2) for multinational leaflets, also a significant difference (p < 0.05). The mean scores for NSAIDs were similar between local (16.6, SD ± 5.9, n = 37) and multinational (16.9, SD ± 5.4, n = 37) companies. For antidepressants, the mean score was 16.5 ± 4.1) for local companies and 17.3 ± 4.3) for multinational companies, showing no significant difference.

## Discussion

The study evaluated the quality of PILs provided by local and multinational pharmaceutical companies in Pakistan using a comprehensive scoring system to assess various quality parameters. The findings indicated significant differences in the quality of PILs between the two groups, with MNCs generally providing higher quality information compared to local companies.

The study reveals that PILs prepared by MNCs offer more specific dosage information that may be useful in different categories of patients. Renal dose adjustments were discussed in 57.1% of multinational PILs compared to 48% of local ones and hepatic dose adjustments were included in 50.9% of multinational PILs compared to 40% of domestic PILs. These findings are consistent with the findings of Eshtayeh et al. who noted that the consideration of dosage adjustment for patients with renal or hepatic problems was more elaborate in PILs of multinational firms [6]. Incorporating information about medication use in hemodialysis patients was also significantly higher in multinational PILs (26.3%) than local PILs (19.6%). Marin et al. (2020) demonstrated that hemodialysis patients are particularly vulnerable to polypharmacy, increasing their risk of adverse drug events and medication errors. In such cases, accurate and comprehensive PILs are critical to ensure safe medication use. However, our results indicate that current PILs—whether from multinational or local manufacturers—frequently lack this vital information. This discrepancy underscores the urgent need for regulatory improvements in PIL standards, especially for medications commonly prescribed to high-risk groups like dialysis patients [11].

PILs for adults are even more different: multinational, 50.1%. have higher indication-specific dosing than local 38.1%. Concerning the dosing information about the age of patients, pediatric and geriatric dosing information represents another tendency of better documentation in multinational PILs. Similar results were also supported by a study stating that more should be done to improve the availability of prescribing information, particularly dosing and safety, on drug labels for older people [12]. Pharmacokinetics and pharmacodynamics factors are also affected by obesity; therefore dosing regimen of obese patients are different from that of normal-weight patients [13]. The studies documented in the literature reveal that PILs rarely provide specific dosing recommendations for obese patients [14]. The current study also revealed that dosing for obese patients referred to only about 10% of PIL for both multinational and local pharmaceutical PILs.

Dysphagia presents a significant medication administration challenge, with 28% of hospitalized patients and 67% of senior housing residents requiring crushed tablets for proper drug delivery [15]. Crushing tablets alters the rate of drug release, resulting in overdose or an under dose, and also destroys the sustained release aspect, which causes the immediate release of the active ingredient. This information is essential to ensure the correct dosing and avoidance of overdose and alter release effects [16]. The present study indicates that, on average, approximately 89% of the details about the fact that a tablet cannot be crushed were omitted in PILs. However, it was statistically significantly more often provided in multinational PILs (13.8%) than in local ones (6.7%). Ishaqui et al. noted the same; the study mentioned that such information was reported only in nine PILs.

In most cases, the multinational PILs provide more detailed information on DDI than the local PILs. Concerning the DDI details, earlier studies show Australian PILs contained 90% while the US PILs have 68% information [17]. Similarly in Palestine, imported NSAIDs brands had more comprehensive DDI data than locally produced ones [5]. In the current study, details on DDI were recorded in 597 (85.1%) of the PILs. Still, this information was reported significantly more frequently in multinational PILs than in local PILs. Black box warnings call attention to crucial safety information so that prescribers and patients are aware of severe and possible fatal reactions or side effects [18]. Beach et al. studied 206 most commonly used brands of drugs in the US. They reported that black box warnings were given in 60% of the PILs [19]. In the current study, black box warnings were mentioned in less than half of the PILs, with a statistically significant higher frequency in multinational PILs compared to local PILs. The information regarding potential side effects and toxicity is often included, but the comprehensiveness and details vary widely [20,21]. The current study’s results revealed that treatment of toxicity was mentioned in only one-third of the PILs, which is low compared to results of George et al. reporting around 60% of PILs containing detailed information on adverse effects and toxicity management [20]. In contrast, another recent study reported that only 40% of PILs included clear indications and management strategies for toxicity, emphasizing the variability in the detail and comprehensiveness of this critical information [21].

Suspected ADRs reporting by the patients can help minimize drug safety risks and strengthen the pharmacovigilance system by providing valuable real-world data. An earlier study found that patient reporting significantly increased the number of ADR reports in some countries, experiencing a 30% increase in total reported reactions over several years [22]. The current study revealed that information about how to report ADRs was missing in around 55% and 62% of multi and local pharmaceutical PILs, respectively, which can be considered as a contributing factorimpacting and resulting in lower ADR reporting [23]. The overall finding revealed considerable disparities among the quality of information mentioned in PIL of multinational and local manufacturers of Pakistan which is similar to the findings of other studies conducted in various countries. [7,24,25]. The current study also noticed that PILs were often in English language which affects the readability and understanding of the essential information, as English is not understood by many consumers. Similar results also have been identified by other research studies so addition of Urdu translation can enhance the understanding of the information provided in PILs [26,27].

The gaps identified in the quality and comprehensiveness of PILs provided by multinational and local drug manufacturers demonstrate the urgent need for systemic changes in the latter’s approach to patient information provision. Although multinational firms have established the standards of detailed and extensive PILs, local firms must follow these examples to provide patients with adequate information to use medicines correctly. Moreover, physician should encourage patients to read the PILs for an effective and safe use of the medication. Pharmacist can help in better use of PILs during dispensing medication to the patients and should assess the patients on any readability queries regarding PILs,

## Conclusion

Disparity is observed in the quality and the extent of information provided in the PILs of Pakistan’s multinational and local pharma companies. The study revealed that multinational companies generally develop higher-quality PILs; therefore, local companies should also improve their informational standards as improving comprehensiveness and user-friendliness of PILs can help local companies enhance patients’ trust and also promote safe medication usage. These improvements are also critical to providing effective and standardized patient education to improve patients’ health status. The study emphasizes on the role of regulatory action and industry initiatives to ensure that all PILs, irrespective of their source, contain the information the patients require for the appropriate use of medicines. It is also recommended to add Urdu translation in PILs for better understanding and assessing of the given information.

## Supporting information

S1 File Supplementary data file. (XLSX)
